# Correlation of Facial Form with Temporomandibular Joint-Space Dimensions: A CBCT-Based Cross-Sectional Study

**DOI:** 10.3390/dj14060326

**Published:** 2026-06-01

**Authors:** Mohammad Gazali, Fuad Husain Akbar, Acing Habibi Mude, Fadhlil Ulum A. Rahman, Babatunde Olamide Bamgbose, Muhammad Ruslin

**Affiliations:** 1 Department of Oral and Maxillofacial Surgery, Faculty of Dentistry, Hasanuddin University, Jl. Perintis Kemerdekaan KM. 10, Makassar 90245, South Sulawesi, Indonesia; mohammadgazali@unhas.ac.id; 2 Hasanuddin Dental Hospital, Jl. Kandea No. 5, Makassar 90157, South Sulawesi, Indonesia; 3 Department of Dental Public Health, Faculty of Dentistry, Hasanuddin University, Jl. Perintis Kemerdekaan KM. 10, Makassar 90245, South Sulawesi, Indonesia; fuadhusainakbar@unhas.ac.id; 4 Department of Prosthodontics, Faculty of Dentistry, Hasanuddin University, Jl. Perintis Kemerdekaan KM. 10, Makassar 90245, South Sulawesi, Indonesia; acinghabibie@unhas.ac.id; 5 Department of Oral and Maxillofacial Radiology, Faculty of Dentistry, Hasanuddin University, Jl. Perintis Kemerdekaan KM. 10, Makassar 90245, South Sulawesi, Indonesia; fadhlilulum@unhas.ac.id; 6 Aminu Kano Teaching Hospital, Zaria Road, Kano 700233, Kano State, Nigeria; drtundebamgbose@yahoo.com

**Keywords:** temporomandibular joint space, facial form, facial morphology, cone-beam computed tomography, CBCT

## Abstract

**Introduction:** The relationship between facial form and temporomandibular joint (TMJ) joint-space dimensions remains unclear, particularly regarding variation across different facial shapes. Cone-beam computed tomography (CBCT) provides accurate three-dimensional evaluation of TMJ structures and allows precise assessment of joint-space dimensions. This study aimed to evaluate the association between facial form and TMJ joint-space dimensions (anterior, superior, and posterior) using CBCT. **Materials and Methods:** This cross-sectional study included 69 adults aged 18–50 years with complete permanent dentition and no signs of temporomandibular disorders based on the Fonseca Index. Facial form was classified as round, oval, or square using standardized two-dimensional photography and an algorithm-based facial classification method (Fisherface-based analysis). CBCT images were obtained using a standardized TMJ protocol, and anterior, superior, and posterior joint spaces were measured bilaterally. Statistical comparisons were performed using ANOVA or Kruskal–Wallis tests (*p* < 0.05). **Results:** Most joint-space dimensions showed no significant differences among facial forms. A significant variation was observed only in the left posterior joint space (*p* = 0.002), where round-faced individuals exhibited the smallest mean value (2.39 ± 1.64 mm) compared with oval (3.77 ± 2.01 mm) and square (6.01 ± 3.12 mm) facial types. **Conclusions:** Facial form demonstrated minimal influence on temporomandibular joint joint-space dimensions, with differences observed only in the left posterior compartment. These findings suggest that facial-shape assessment may have limited but potentially complementary value in TMJ evaluation. However, the clinical implications should be interpreted cautiously given the predominantly non-significant findings.

## 1. Introduction

Temporomandibular disorders (TMDs) encompass a group of musculoskeletal conditions involving the temporomandibular joint (TMJ), associated musculature, and related structures. These disorders commonly present as joint pain, limited mandibular motion, and TMJ sounds, significantly affecting quality of life [1]. The multifactorial etiology of TMD includes anatomical, functional, psychosocial, and occlusal components, with increasing attention being given to craniofacial morphology as a potential risk factor [2].

Facial morphology has become an increasingly important component of comprehensive dental treatment planning, as facial characteristics influence clinical decision-making in prosthodontics, orthodontics, and implantology. Recent digital workflows integrating three-dimensional facial scans with CBCT and intraoral data have enhanced virtual patient analysis and interdisciplinary treatment planning, highlighting the clinical relevance of facial analysis beyond esthetic considerations and supporting its integration into temporomandibular joint research [3,4,5].

Several studies have reported associations between skeletal growth patterns and variations in TMJ joint-space dimensions, particularly with vertical facial patterns. Hyperdivergent individuals tend to exhibit smaller condyles, larger joint spaces, and different condyle–fossa relationships compared to hypodivergent and normodivergent counterparts. These findings suggest that vertical skeletal discrepancies may have a role in predisposing individuals to altered joint biomechanics [6].

Recent evidence shows that the facial form classification methods provide a reliable approach for categorizing facial morphology. Functional studies also demonstrate that individuals with temporomandibular disorders, particularly those with jaw-muscle pain, exhibit reduced masticatory efficiency and altered muscle performance compared with healthy controls. Although these findings do not directly link facial index categories with muscle activity, they suggest that variations in facial morphology may influence masticatory function and susceptibility to muscle-related TMD [7,8,9].

Although the facial index is well established as an objective method for classifying facial morphology and documenting variation in craniofacial proportions, recent CBCT-based investigations of the temporomandibular joint have focused mainly on evaluating joint-space changes in pathological conditions such as juvenile idiopathic arthritis rather than on differences across facial forms. As a result, the relationship between soft-tissue facial form, as measured through the facial index, and hard-tissue TMJ joint-space dimensions including joint-space dimensions assessed through CBCT remains insufficiently explored [7,8,9].

Therefore, the present study aims to evaluate the association between facial form and temporomandibular joint joint-space dimensions (anterior, superior, and posterior) using CBCT in asymptomatic adult individuals. It is hypothesized that certain facial forms may exhibit distinct TMJ morphometric profiles, potentially serving as early indicators of structural vulnerability to dysfunction.

## 2. Materials and Methods

### 2.1. Study Design and Ethical Approval

This analytic cross-sectional study evaluated the relationship between facial shape and temporomandibular joint (TMJ) joint-space dimensions. Ethical approval was granted by the Health Research Ethics Committee of the Faculty of Dentistry and Hasanuddin University Dental Hospital, Universitas Hasanuddin. The study was reviewed as Exempted (minimal risk) and approved under Ethical Approval Number 0075/PL.09/KEPK FKG–RSGM UNHAS/2024, Protocol Number UH17121037, valid from 24 January 2024 to 24 January 2025.

All procedures adhered to the Declaration of Helsinki, and written informed consent was obtained from all participants prior to enrollment.

### 2.2. Participants

A total of 69 adults aged 18–50 years were included.

Inclusion criteria:

1. Complete permanent dentition (excluding third molars).
2. No history of orthodontic treatment.
3. Absence of temporomandibular disorder (TMD) symptoms based on the Fonseca Anamnestic Index.

Exclusion criteria:

1. Craniofacial anomalies.
2. History of maxillofacial trauma.
3. Systemic musculoskeletal disorders.
4. Incomplete CBCT records.

### 2.3. Facial Shape Measurement

Facial shape was assessed using standardized 2D frontal facial photographs taken with a Canon EOS 450D camera (Canon Inc., Tokyo, Japan) and 50-mm lens under controlled lighting and a neutral background. Participants were positioned in Natural Head Position with the Frankfurt Horizontal Plane parallel to the floor, relaxed facial musculature, and teeth in centric occlusion.

Facial-shape classification was performed using the Fisherface algorithm implemented in Python (OpenCV, version 4.8.0), which applies linear discriminant analysis (LDA) to extract global facial features and categorize facial morphology. Based on this automated pattern-recognition method, participants were categorized into three groups:

- Round face
- Oval face
- Square face

### 2.4. CBCT Imaging Protocol

All participants underwent cone-beam computed tomography (CBCT) examination using a Vatech CBCT system with a field of view (FOV) of 170 × 150 mm. CBCT imaging was performed in this asymptomatic population to enable accurate three-dimensional evaluation of osseous temporomandibular joint morphology and joint-space dimensions, including subtle anatomical variations and side-related asymmetries that may not be detectable through clinical examination alone. Scans were obtained with the Frankfurt Horizontal Plane parallel to the floor and the mandible positioned in centric occlusion.

Image acquisition followed the standard TMJ preset protocol of the Vatech unit, in which exposure parameters (kVp, mA, and exposure time) and voxel resolution are automatically determined by the manufacturer. The voxel size for TMJ imaging was set at 0.3 mm, which is commonly used for high-resolution assessment of TMJ structures. CBCT datasets were reconstructed and analyzed using EzDent-I software version 3.5.0.4 (Vatech Co., Ltd., Hwaseong, Republic of Korea), allowing standardized multiplanar sagittal orientation through the central axis of the condyle.

### 2.5. TMJ Morphological Measurements

TMJ joint-space dimensions were measured bilaterally using the linear measurement tools in EzDent-I. The following measurements were recorded for each TMJ:

- Anterior joint space
- Superior joint space
- Posterior joint space

**Figure 1:**
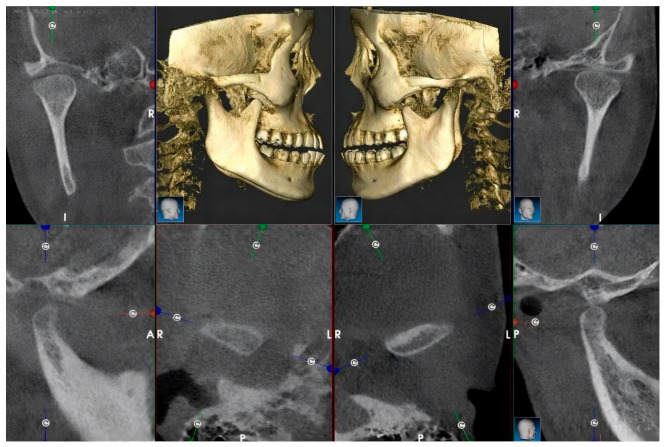
Sagittal CBCT images demonstrating standardized measurement of TMJ joint spaces.

All joint-space measurements (anterior, superior, and posterior) were defined as linear distances between the condylar surface and the corresponding points of the glenoid fossa in the sagittal plane (Figure 1).

Measurements were obtained by two blinded observers, and intra- and inter-observer reliability were confirmed, with an intraclass correlation coefficient (ICC = 0.93), indicating excellent measurement reliability.

Sagittal cone-beam computed tomography (CBCT) views showing the measurement of the anterior, superior, and posterior joint spaces of the temporomandibular joint (TMJ). Measurements were obtained bilaterally with the mandible in centric occlusion using EzDent-I software. Linear distances were recorded from the condylar surface to the corresponding points of the glenoid fossa following a standardized multiplanar reconstruction protocol.

### 2.6. Statistical Analysis

Normality of data distribution was assessed using the Shapiro–Wilk test. Depending on data characteristics, ANOVA or Kruskal–Wallis tests were employed to compare joint-space dimensions across facial-shape groups. When significant differences were detected, post hoc analyses with Bonferroni or Dunn correction were applied to control for multiple comparisons where appropriate. Statistical significance was set at *p* < 0.05. The statistical approach was selected based on the study design and data distribution, focusing on group comparisons rather than predictive or multivariate modeling. Right and left TMJ measurements were analyzed separately to allow evaluation of potential side-related differences and asymmetry.

## 3. Results

A total of 69 participants were included in the analysis. The majority presented with an oval facial form (59.4%), followed by round (20.3%) and square (20.3%) facial types. Across all participants, the mean TMJ joint-space values ranged between 3.03 mm and 4.90 mm for anterior and superior spaces, with wider variability observed in posterior spaces.

### 3.1. Comparison of Joint-Space Dimensions Across Facial Forms

Table 1 summarizes the anterior, superior, and posterior joint-space dimensions for the right and left TMJs. No significant differences were detected among facial-shape groups for the anterior (right: *p* = 0.826; left: *p* = 0.434) or superior joint spaces (right: *p* = 0.453; left: *p* = 0.507). Posterior joint-space values on the right side were also not significantly different (*p* = 0.151).

A statistically significant difference was observed for the left posterior joint space (*p* = 0.002). Post hoc analysis showed that square-faced individuals exhibited a larger left posterior joint space compared with round-faced individuals, with a large effect size (Cohen’s d = 1.50). Round-faced individuals demonstrated the smallest mean posterior joint space (2.39 ± 1.64 mm), followed by oval (3.77 ± 2.01 mm), whereas square-faced subjects exhibited the largest posterior joint space (6.01 ± 3.12 mm). Post hoc analysis indicated that square-faced individuals differed significantly from both round- and oval-faced groups.

### 3.2. Visualization of Joint-Space Variability

**Figure 2:**
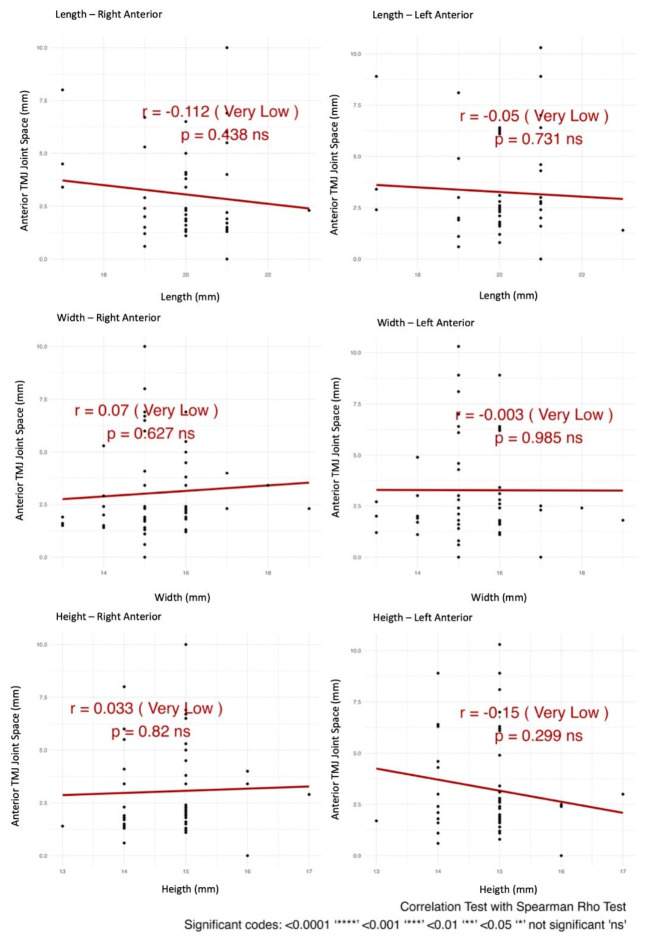
Boxplots illustrating anterior, superior, and left posterior temporomandibular joint-space dimensions by facial form.

Figure 2 illustrates boxplots for each joint-space parameter by facial form. The distribution patterns visually confirm the absence of group differences for anterior and superior compartments and highlight the marked separation in left posterior joint-space values, particularly the wider dispersion among square-faced subjects.

## 4. Discussion

The present study evaluated the association between facial morphology and temporomandibular joint (TMJ) joint-space dimensions using cone-beam computed tomography (CBCT), which provides high-resolution three-dimensional assessment of osseous joint structures. Given the exploratory nature of this study, the statistical analysis was designed to identify group differences rather than establish predictive relationships. Oval facial types constituted the majority of the sample, followed by round and square forms. While no statistically significant differences were observed in anterior and superior joint spaces among facial types, a significant reduction in the left posterior joint space was identified in round-faced individuals (*p* = 0.002).

CBCT remains the preferred imaging modality for TMJ evaluation due to its ability to detect subtle variations in condylar morphology, joint spaces, and asymmetries that may not be apparent on two-dimensional imaging. Previous studies have reported associations between posterior joint-space narrowing and internal derangements, particularly anterior disc displacement, which is consistent with the reduced posterior joint space observed in round-faced participants in the present study. In addition, reduced joint-space dimensions have been reported in individuals with diminished occlusal support, suggesting that condylar position may be responsive to functional loading patterns; however, this interpretation should be considered cautiously. Overall, most joint-space dimensions did not show significant differences among facial forms, indicating a limited influence of facial morphology in this sample [10,11].

Evidence from craniofacial and functional studies indicates that masticatory muscle morphology and activity vary according to facial type. Broad- or short-face patterns have been associated with increased muscle thickness, stronger elevator muscle activity, and higher bite-force capacity compared with long-face patterns [12,13,14]. It is hypothesized that these functional characteristics may be associated with altered posterior condylar loading; however, this interpretation remains speculative and should be interpreted cautiously, as muscle activity and bite force were not directly measured in the present study. Similar associations between broader facial morphology and increased severity of TMJ dysfunction have been reported previously [15].

Differences in muscle strength and craniofacial structure may also influence TMJ biomechanics and condylar positioning. Broader facial patterns have been linked to increased masseter muscle thickness and volume, which may alter joint loading [12,16,17]. Nevertheless, not all craniofacial variations lead to temporomandibular disorders, and skeletal configurations such as mandibular retrognathia, hypodivergent growth patterns, and asymmetry appear to play a more prominent role in predisposing individuals to degenerative TMJ changes [18].

Mandibular asymmetry has been shown to affect condylar morphology and joint-space dimensions, reflecting altered biomechanical behavior of the TMJ. Three-dimensional CBCT studies have demonstrated measurable side-related differences in individuals with craniofacial asymmetry, and similar patterns have been associated with TMD symptoms in younger populations [19,20]. The ability of CBCT to detect posterior joint-space asymmetry in the present study further supports its relevance for comprehensive TMJ assessment.

Previous studies have suggested potential associations between facial morphology and muscular TMD; however, such relationships were not directly evaluated in the present study. Individuals with broader facial forms have been reported to constitute a higher proportion of myofascial pain cases, indicating a potential relationship between facial proportion, muscle function, and TMJ kinematics [21]. The present findings parallel these observations, as narrower posterior joint spaces were identified in round-faced individuals, whereas square-faced participants demonstrated larger posterior joint-space values, which may reflect adaptive condylar positioning rather than pathological change [15].

Finally, population-specific normative data highlight the importance of considering ethnic and morphological variation when interpreting craniofacial measurements and TMJ joint-space dimensions. CBCT continues to be recognized as a reliable tool for identifying joint-space alterations, condylar remodeling, and early degenerative changes that may not yet be clinically evident [22]. Overall, the observed association between facial morphology and posterior joint-space asymmetry may suggest a potential association between facial type and TMJ biomechanics; however, this finding should be interpreted cautiously given the predominantly non-significant results.

Although the present study demonstrated minimal influence of facial form on TMJ joint-space dimensions, aside from the left posterior space, these findings should be interpreted within the limitations of two-dimensional facial classification. Future studies incorporating three-dimensional facial analysis and quantitative assessment of condylar volume may provide a more comprehensive understanding of the relationship between facial morphology and temporomandibular joint biomechanics and may reveal subtler associations not captured by joint-space dimensions alone.

## 5. Limitations

This study has several limitations. Its cross-sectional design prevents causal interpretation, and the facial-shape assessment based on two-dimensional photographs may not fully capture underlying skeletal morphology. CBCT provided detailed osseous evaluation but did not assess soft-tissue components such as disc position or muscle activity. The uneven distribution of facial types may also limit generalizability.

The statistical analysis was limited to group comparisons and did not include multivariate or regression modeling, which may reduce the ability to account for potential confounding factors. Furthermore, bilateral TMJ measurements were analyzed separately, which may not fully account for within-subject correlation and should be considered when interpreting the results.

## 6. Conclusions

This study demonstrated that facial shape has minimal influence on most TMJ joint-space dimensions, with a significant difference observed only in the left posterior compartment, where round-faced individuals exhibited the narrowest mean space. These findings suggest that posterior joint-space variation may reflect localized biomechanical adaptations related to facial morphology. Clinically, incorporating facial-shape assessment into TMJ evaluation may help identify individuals who could be predisposed to altered condylar loading. Future studies integrating muscle activity analysis and three-dimensional morphological assessment are recommended to further clarify these associations.

## Figures and Tables

**Table 1:** Comparison of TMJ joint-space dimensions (mean ± SD, mm) among facial forms.

Facial Dimension	TMJ Side	r Value	*p* Value	Correlation Strength	Significance
Facial length	Dextra	−0.112	0.438	Very low	Not significant (ns)
	Sinistra	−0.050	0.731	Very low	Not significant (ns)
Facial width	Dextra	0.070	0.627	Very low	Not significant (ns)
	Sinistra	−0.003	0.985	~0 (no correlation)	Not significant (ns)
Facial height	Dextra	0.033	0.820	Very low	Not significant (ns)
	Sinistra	−0.150	0.299	Very low	Not significant (ns)

## Data Availability

The data presented in this study are available on request from the corresponding author. The data are not publicly available due to privacy and ethical restrictions involving human participants.
